# Molecular epidemiology of *Acinetobacter baumannii* in different hospitals in Tripoli, Lebanon using *bla*_OXA-51-like_ sequence based typing

**DOI:** 10.1186/s12866-015-0441-5

**Published:** 2015-05-16

**Authors:** Rayane Rafei, Hélène Pailhoriès, Monzer Hamze, Matthieu Eveillard, Hassan Mallat, Fouad Dabboussi, Marie-Laure Joly-Guillou, Marie Kempf

**Affiliations:** ATOMycA, Inserm Atip-Avenir Team, CRCNA, Inserm U892, 6299 CNRS, University and Centre Hospitalier Universitaire d’Angers, Angers, France; Laboratoire Microbiologie Santé et Environnement (LMSE), Centre AZM pour la recherche en Biotechnologie et ses applications, Université Libanaise, Tripoli, Lebanon; Laboratoire de Bactériologie, Institut de Biologie en Santé - PBH, CHU, 4 rue Larrey, 49933 Angers Cedex, France

**Keywords:** *Acinetobacter baumannii*, Lebanon, Carbapenem resistance, *bla*_OXA-51_ sequence based typing, MLST, OXA-23, NDM-1

## Abstract

**Background:**

*A. baumannii* has emerged as an important nosocomial pathogen with an outstanding ability to acquire multidrug resistant mechanisms. In this study, we investigate the molecular epidemiology and carbapenem resistance mechanisms of *A. baumannii* in Tripoli, Northern Lebanon.

**Methods:**

One hundred sixteen non-duplicate isolates isolated between 2011 and 2013 in different hospitals in Tripoli, Lebanon from Lebanese patients and wounded Syrian patients during Syrian war were studied. Antibiotic susceptibility testing was determined by agar disc diffusion and Etest. Carbapenemase-encoding genes were investigated by PCR. All isolates were typed by *bla*_OXA-51-like_ sequence based typing (SBT) and 57 isolates were also analysed by MLST using Pasteur’s scheme followed by eBURST analysis.

**Results:**

Of the 116 isolates, 70 (60 %) showed a carbapenem resistance phenotype. The *bla*_OXA-23_ with an upstream insertion of IS*Aba1* was the major carbapenem resistance mechanism and detected in 65 isolates. Five isolates, including four from wounded Syrian patients and one from a Lebanese patient, were positive for *bla*_NDM-1_. *bla*_OXA-51-like_ SBT revealed the presence of 14 variants, where *bla*_OXA-66_ was the most common and present in 73 isolates, followed by *bla*_OXA-69_ in 20 isolates. MLST analysis identified 17 sequence types (ST) and showed a concordance with *bla*_OXA-51-like_ SBT. Each clonal complex (CC) had a specific *bla*_OXA-51-like_ sequence such as CC2, which harboured *bla*_OXA-66_ variant, and CC1 harbouring *bla*_OXA-69_ variant. NDM-1 producing isolates belonged to ST85 (4 Syrian isolates) and ST25 (1 Lebanese isolate).

**Conclusions:**

Our results showed a successful predominance of international clone 2 with a widespread occurrence of OXA-23 carbapenemase in Lebanese hospitals. These findings emphasise the urgent need of effective measures to control the spread of *A. baumannii* in this country.

## Background

*A. baumannii* is an opportunistic pathogen mainly involved in healthcare-associated infections, with increased mortality and morbidity [1]. The serious concern associated with this bacterium is the increasing prevalence of multidrug resistant isolates, especially carbapenem resistant ones [2]. Therefore, management of infections due to *A. baumannii* has become a real public health issue in many countries [3]. In a recent SENTRY Antimicrobial Surveillance program, the percentage of *A. baumannii* isolates susceptible to meropenem decreased noticeably from 52.9 % in 2009 to 37.7 % in 2011 in European intensive care units [4]. The main mechanism of resistance to carbapenems is the production of oxacillinases encoded mostly by *bla*OXA-23-like, *bla*OXA-24-like, *bla*OXA-58-like and recently *bla*OXA-143-like and *bla*OXA-235-like [2, 5–7]. To a lesser degree, carbapenemases from classes A and B have also been involved [2, 5]. New Delhi metallo-ß-lactamase 1 (NDM-1), a recent described metallo-ß-lactamase, has emerged in *Enterobacteriaceae* as well as in *Acinetobacter* [8]. Generally, a limited number of clones are responsible for worldwide outbreaks, and international clones I and II are the most common ones [9]. In order to identify and track these clones during hospital outbreaks, numerous molecular typing methods have been proposed such as pulsed-field gel electrophoresis (PFGE), amplified fragment length polymorphism (AFLP), repetitive-sequence-based PCR (rep-PCR), and multilocus sequence typing (MLST) [10–14] . Currently, MLST is the gold standard typing tool for population structure and macro-epidemiological investigations based on sequencing of internal fragments of 7 genes [14]. Two MLST schemes have been developed for *Acinetobacter baumannii* : Pasteur and Bartual schemes [10, 15]. Overall, the 2 schemes are concordant [14]. However, because of recombination bias observed on some loci in Bartual’s MLST (*gyrB* and *gpi*) [16, 17], Pasteur’s MLST has been chosen for this study. Recently, *bla*OXA-51-like sequence-based typing (SBT), a simple typing method based on sequencing of the full length of *bla*OXA-51-like gene has been proposed as it has shown a similar discriminatory power than rep-PCR, and MLST [16, 18–20].

In Lebanon, limited numbers of epidemiological studies concerning *A. baumannii* have been reported, almost in Beyrouth [20–23]. In a previous study, we have described the dominance of international clone II within a set of 42 isolates collected from Beyrouth and North Lebanon between 2009–2012 [20]. In another study, we reported the first detection of four NDM-1 producing isolates isolated in Tripoli-Northern Lebanon from Syrian patients injured during Syrian War [24]. The aim of this study was to characterise *A. baumannii* molecular epidemiology in Tripoli-Lebanon between 2011–2013 using *bla*OXA-51-like and Pasteur’s MLST typing and to determine the carbapenem resistance mechanisms.

## Results

### Bacterial isolates and identification

A total of 116 non-duplicate isolates were obtained between 2011 and 2013 from seven medical institutions in Tripoli: 73 isolates from Tripoli Governmental hospital, 34 from Nini hospital, four from Dar Al Chiffaa hospital, two from Monla, one isolate from each Hanane and Haykal hospitals and one from the private laboratory. Among those isolates, 59 had been obtained from Syrian refugees and 57 from Lebanese patients. They were all confirmed as *A. baumannii* by *bla*_OXA-51_ real time PCR and *rpoB* gene sequencing. These isolates were recovered from respiratory specimens (25/116; 21.6 %); wound (68/116; 58.6 %); urine (10/116; 8.6 %); catheter tips (8/116; 6.9 %), blood (1/116; 0.9 %) and other locations (3/116; 2.6 %). For one isolate, no information was available. Ninety patients were male and 26 female. Ages were between 1 month and 89 years with a mean of 40 years.

### Antibiotic susceptibility and carbapenem resistance mechanisms

Most of the isolates exhibited multidrug-resistant phenotypes. Overall, 70 isolates were carbapenem resistant and among them, 65 carried the *bla*_OXA-23_ gene while 5 isolates carried the *bla*_NDM-1_ gene. IS*Aba1* was found in 101 isolates. The presence of IS*Aba1*/*bla*_OXA-23_ association was confirmed in all OXA-23 producing isolates. The IS*Aba1*/*bla*_OXA-51_ association was not detected. Among carbapenem-resistant *A. baumannii*, 43 had been isolated from Syrian refugees and 27 from Lebanese patients. The prevalence of carbapenem resistance was significantly higher among *A. baumannii* isolated from Syrian refugees (74 % vs. 47 %; *P* < 0.01).

### Epidemiological typing

The *bla*_OXA-51-like_ SBT revealed the presence of 14 different nucleotide variants. Of the 116 isolates, *bla*_OXA-66_ variant was the most prevalent and found in 73 isolates, followed by *bla*_OXA-69_ (20 isolates), *bla*_OXA-64_ (7 isolates)*, bla*_OXA-94_ (5 isolates) and *bla*_OXA-120_ (2 isolates). Other variants, *bla*_OXA-70_, *bla*_OXA-71_, *bla*_OXA-104_, *bla*_OXA-121_, *bla*_OXA-132_*, bla*_OXA-406_, KF048914, KJ584924, and AKAS01000012 were present sporadically in our collection. *bla*_OXA-406_ variant encoded a new protein (OXA-406) described for the first time in this study which differed from OXA-106 protein by a single amino acid at position 146 (lysine instead of asparagine).

**Fig. 1:**
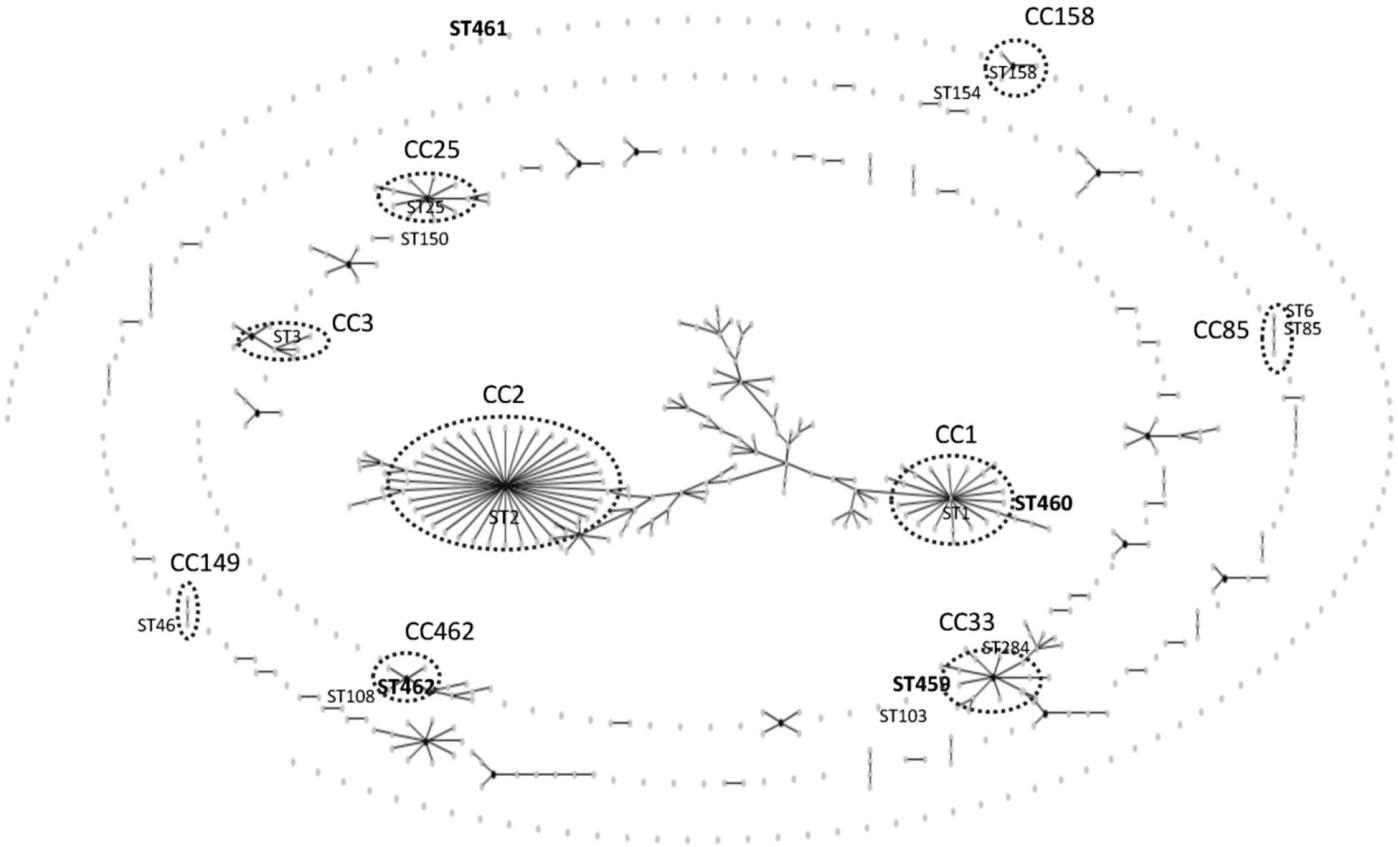
Population snapshot analysed by eBURST on 634 sequences present in MLST Pasteur database (last update 22.10.2014). Red circle showed identified sequence types in this study

MLST was performed on a set of 57 randomly selected *A. baumannii* isolates representing all *bla*_OXA-51_ variants. A total of 17 ST(s), including 4 new ST(s), were identified (Table 1, Fig. 1). eBURST analysis showed that 12 of the 17 ST(s) were clustered into 9 CCs which were CC1, CC2, CC3, CC25, CC33, CC85, CC149, CC158, CC462. Additionally, ST108, ST150, ST154 were single locus variants of ST112, ST444 and ST611 respectively, and ST103 and ST461 were singletons.

**Table 1:** Characteristics of the *Acinetobacter baumannii* isolates studied

No. of isolates	*bla*_OXA-51_ variant	ST* (No. of tested strains)	CC	IMP R	Hospitals (No. of isolates)	Origin of isolation	Nationality	IMP R mechanism
73	*bla*_OXA-66_	ST2 (21)	CC2	56R	TGH (50), Nini (14), Monla (2), private laboratory (1), Haykal (1), Dar Al Chiffaa (4), Hanane (1).	Respiratory specimen (18), catheter tip (7), wound (44), urine (1), other (2), not informed (1)	Syria (43)	IS*Aba1*/OXA-23
							Lebanon (30)	
20	*bla*_OXA-69_	ST1 (12)	CC1	9R	TGH (17), Nini (3)	Respiratory specimen (1), Wound (19)	Syria (11)	IS*Aba1*/OXA-23
		**ST460** (2)					Lebanon (9)	
5	*bla*_OXA-94_	ST85 (4)	CC85	4R	TGH (5)	Wound (5)	Syria (4)	NDM-1
		ST6 (1)					Lebanon (1)	
7	*bla*_OXA-64_	ST25 (6)	CC25	1R	Nini (7)	Respiratory specimens (5), blood (1), urine (1)	Lebanon (7)	NDM-1
1	*bla*_OXA-70_	ST103 (1)	Singleton	S	Nini	Urine	Lebanon	−
1	KF048914	ST154 (1)	C	S	Nini	Urine	Lebanon	−
1	*bla*_OXA-71_	ST3 (1)	CC3	S	Nini	Urine	Lebanon	
1	KJ584924	ST158 (1)	CC158	S	Nini	Urine	Lebanon	−
1	*bla*_OXA-104_	ST46 (1)	CC149	S	Nini	Urine	Lebanon	−
2	*bla*_OXA-120_	**ST459** (1)	CC33	S	Nini	Respiratory specimen (1), urine (1)	Lebanon	−
		ST284 (1)						
1	*bla*_OXA-121_	ST150 (1)	C	S	Nini	Ear	Lebanon	−
1	*bla*_OXA-132_	ST108 (1)	C	S	Nini	Urine	Lebanon	
1	***bla***_**OXA-406**_	**ST461** (1)	Singleton	S	TGH	Catheter tips	Syria	−
1	AKAS01000012	**ST462** (1)	CC462	S	Nini	Urine	Lebanon	−

CC : clonal complex; C : complex formed by two sequence types (no number was assigned because no more of two sequences have been identified yet); IMP : imipenem, ST : sequence type, R : resistance and S : susceptible. Bold type indicates a new ST or a new *bla*_OXA-51_ allele

Comparison between MLST and *bla*_OXA-51-like_ SBT showed that isolates belonging to the same clonal complex carried all the same *bla*_OXA-51_ variant. Besides, each of the remaining lineages (single locus variants or singletons) that were identified had additionally a specific *bla*_OXA-51-like_ variant (Table 1).

## Discussion

Our study provided a global view of molecular epidemiology of *A. baumannii* isolates from hospitals in Tripoli, Lebanon. Tripoli is a city that hosts a large number of refugees and wounded Syrians*,* which may explain the high number of wound specimens in our collection. Although, *A. baumannii* is an opportunistic nosocomial pathogen, it is increasingly reported from infections occurring outside hospitals, particularly skin and soft-tissue infections and pneumonia [25]. *A. baumannii* has also been a well-documented pathogen associated with wound infections in USA troops from the Iraq and Afghanistan wars [26, 27]. The typology of these situations raised questions about their origins and the potential involvement of an environmental reservoir [25]. The origin of infections from Syrian patients was ambiguous. Although it was likely that nosocomial infections occurred, a number of infections were present at the time of admission of the patients in Lebanese hospitals [24]. These infections may have been acquired from environmental sources at the time of injury, during the patient stay in Syrian hospitals, or during a direct evacuation from the theatre of operations to Lebanon. Unfortunately, data concerning the date of injury, the length of hospitalisation, and the conditions of care and treatment in Syrian hospitals are lacking.

Approximately 60 % of our isolates showed a carbapenem resistance phenotype. The production of OXA-23 was the major carbapenem resistance mechanism, with an upstream insertion of IS*Aba1*, thus supporting international data about the worldwide emergence of this carbapenemase [28–30]. Alarmingly, 5 NDM-1 producing isolates have been detected from 4 Syrian patients [24] and one from a Lebanese patient.

*bla*_OXA-51-like_ is an intrinsic oxacillinase gene naturally occurring in *A. baumannii*, and more than 95 variants have been identified to date [31]. However, the occasional detection of *bla*_OXA-51_ in *Acinetobacter nosocomialis* and *Acinetobacter* genomic species “close to 13TU” limit its use as a single identification method. Therefore, it is always combined with others techniques [32, 33]. Genetic diversity of *bla*_OXA-5_ gene has been explored and found to be very interesting for identifying epidemic clones [16, 18, 19, 34, 35]. Thus, the full gene sequencing has been proposed as a single typing method for *A. baumannii*. As being simple and rapid, we have initially screened here the epidemiological belonging of our isolates to the previous identified clonal lineages by *bla*_OXA-51-like_ SBT. We have then randomly selected isolates from each of the representative *bla*_OXA-51-like_ sequences for MLST analysis. In our study, *bla*_OXA-51-like_ gene sequencing correctly assigned all isolates to their corresponding clonal complexes. Here, the *bla*_OXA-66_ gene variant, which is associated with ST2 (belonging to CC2) [16, 19, 35, 36], was predominant and found in 63 % of the *A. baumannii* isolates. The isolates carrying the *bla*_OXA-66_ gene variant have been identified in samples obtained from the different hospitals in Tripoli. These findings are consistent with those observed worldwide since CC2 was reported in more than 34 countries in Europe, Asia, Africa, Australia, USA, and South America [36]. Eighty percent of our carbapenem resistant isolates belonged to this clone and OXA-23 was the only carbapenem resistance mechanism found. The high level of antimicrobial resistance may represent one of the main causes for its propensity and its successful predominance in hospitals throughout the world [10, 36]. The *bla*_OXA-69_ gene variant was the second gene mostly found in our collection. This gene was commonly associated with ST1 (belonging to CC1) but interestingly, we found two *bla*_OXA-69_ isolates belonging to the new sequence type ST460 (SLV of ST1). Both isolates were isolated from wounded Syrian patients. The *bla*_OXA-94_ was another *bla*_OXA-51-like_ gene variant found in our study, it was associated with clonal complex CC85 (named as CC6 in the study of Pournaras *et al*. [19]). CC85 is currently formed by ST6, ST85, ST464, and ST528. In our study *bla*_OXA-51-like_ SBT revealed the same sequence *bla*_OXA-94_ variant in the two identified sequences ST6 and ST85. We have recently detected ST464 in chicken and we found *bla*_OXA- 94_ as a *bla*_OXA-51_ variant [37]. Our 4 NDM-1 producing Syrian isolates belonged to ST85. Indeed, this ST has also been responsible for an outbreak in France. It is usually imported from North Africa [38, 39] and seems to be an emerging clone.

*bla*_OXA-64_ is a *bla*_OXA-51-like_ variant associated with CC25 [19]. ST25 (the founder of CC25) was also an emerging clone reported in Europe, Asia, Africa and USA [36, 40], and from pets in Reunion Island, a French territory located in Western Indian Ocean [41].

Different carbapenemases have been identified in this clone, as OXA-23, OXA-24 and OXA-58 [36]. Otherwise, NDM-1 producing ST25 was also detected in Germany and Kenya [41–43]. Within our four identified isolates belonging to ST25, one isolate was a NDM-1 producing isolate recovered from a urine sample of an 80-year-old Lebanese patient suffering from amyloidosis and anaemia at Nini hospital, Tripoli, Lebanon. Finally, compared to carbapenem resistant isolates belonging to successful emerging clones, most of susceptible isolates were very diverse, belonging to different sequence types (Table 1).

## Conclusions

In conclusion, this study highlights the emergence of NDM-1 and OXA-23 carbapenemases in Tripoli, Lebanon and the urgent need of effective measures to control the spread of *A. baumannii* in this country. It is noteworthy that Tripoli is located near the Syrian border and the microbial epidemiology is probably highly impacted by wounded Syrian refugees who can represent a reservoir of multidrug-resistant bacteria in hospitals. The higher prevalence of carbapenem-resistant *A. baumannii* among isolates from Syrian refugees was consistent with this hypothesis. Besides, we showed that *bla*_OXA-51-like_ SBT is a reliable and fast method able to assign our isolates to their clonal complexes. Other multicentre studies are required to investigate the situation in other Lebanese cities that are possibly less concerned by the afflux of Syrian refugees fleeing war.

## Methods

### Bacterial isolates and Identification

The *A. baumannii* clinical isolates used in this study were collected between 2011 and 2013 from microbiological laboratories covering 6 hospitals in Tripoli, Lebanon: Tripoli Governmental hospital (100 beds), Nini (120 beds), Dar Al Chiffaa (120 beds), Monla (120 beds), Hanane (45 beds), and Haykal (125 beds) hospitals and one private laboratory. Isolates were sent to Azm Center for Research in Biotechnology and its application and stored at-80 °C. This study was also approved by the ethical committee of Azm center under the authorization N° 07/2012. Bacterial identification was initially performed by matrix assisted laser ionization time of flight mass spectrometry (MALDI-TOF MS) using the Vitek®MS (bioMérieux, Marcyl’Étoile, France) and the identification of *A. baumannii* at species level was confirmed by real time PCR of the *bla*_OXA-51_ gene [44] and partial RNA polymerase ß-subunit (*rpoB*) gene sequencing [45].

### Susceptibility testing and detection of carbapenemases

Antibiotic susceptibility testing was performed by the disc diffusion method according to the recommendations of the French Society of Microbiology (www.sfm-microbiologie.org/). A panel of 14 antibiotics was tested including ticarcillin, ticarcillin/clavulanic acid, piperacillin/tazobactam, ceftazidime, imipenem, ciprofloxacin, amikacin, gentamicin, tobramycin, trimethoprim/sulfamethoxazole, colistin, netilmicin, doxycycline and rifampicin. Carbapenem resistance was confirmed by determining minimum inhibitory concentration (MICs) against imipenem, meropenem and doripenem by Etest®strips (bioMérieux). Carbapenem resistant isolates were investigated by PCR assays for the presence of carbapenemase-encoding genes *bla*_OXA-23_ [46], *bla*_OXA-24_ [46], *bla*_OXA-58_ [46], *bla*_OXA-143_ [6], *bla*_NDM-1_ [47], *bla*_IMP_ [5], *bla*_VIM_ [5], *bla*_KPC_ [48] and the insertion sequence IS*Aba1* [49]. The presence of IS*Aba1* upstream *bla*_OXA-23_ or *bla*_OXA-51_ genes was searched using a combination of IS*Aba1* primers with reverse primers of *bla*_OXA-23_ or *bla*_OXA-51_ respectively.

### Epidemiological typing

Epidemiological typing of all isolates was carried out by *bla*_OXA-51-like_ sequence based typing (SBT) as described previously [16]. The sequences were compared to all variants present in BLAST. Each new sequence detected was submitted to GenBank and assigned by Lahey database for beta-lactamase classification (http://www.lahey.org/studies/webt.asp). MLST was performed on a panel of 57 isolates according to the Pasteur scheme (http://www.pasteur.fr/mlst). When a new allele or a new sequence type (ST) was identified, it was submitted to Pasteur Institute MLST Database. eBURST (http://eburst.mlst.net/) was applied to compare identified ST(s) to available ST(s) present in MLST Database (last update 22.10.2014). A clonal complex (CC) was defined as a set formed by the founder ST and its single locus variants (SLV) [10].

### Nucleotide sequence accession numbers and novel sequence types

Four newly identified ST(s) have been coded by MLST Pasteur as ST459, ST460, ST461 and ST462. A new nucleotide variant of *bla*_OXA-51_ was submitted to GenBank under the submission number [GenBank: KJ584915] and assigned by Lahey center as *bla*_OXA-406_.

### Statistical analysis

The comparison of the prevalence of carbapenem-resistant *A. baumannii* in isolates from Syrian refugees and in isolates from Lebanese patients was performed with the Chi-square for test. A *P* < 0.05 was considered significant.
